# Tuberculosis vaccination: microbiological and immunological summary of a series of experimental challenge studies

**DOI:** 10.1186/s13567-025-01637-2

**Published:** 2025-10-24

**Authors:** Ramon A. Juste, Iker A. Sevilla, Esmeralda Minguijon, Miguel Fuertes, Natalia Elguezabal, Mariví Geijo, Patricia Vazquez, Miriam Serrano, Arrazuria Rakel, Christian Gortazar, Amaia Etxezarreta, Elena Molina, Joseba M. Garrido

**Affiliations:** 1 NEIKER-Instituto Vasco de Investigación y Desarrollo Agrario. Animal Health Department. Derio, Bizkaia, Spain; 2 Department of Parasitology, Veterinary School, Universidad de Leon, Campus de Vegazana, s/n., Leon, Spain; 3 SaBio (Health and Biotechnology), Instituto de Investigación en Recursos Cinegéticos IREC (CSIC-UCLM), Ciudad Real, Spain

**Keywords:** Tuberculosis, cattle, skin test, interferon gamma, infection, transmission, vaccine, inactivated vaccine, *M. bovis*, route

## Abstract

The complexity of bovine tuberculosis (TB) epidemiology, especially in extensive livestock systems with wildlife reservoirs, calls for novel control strategies such as vaccination. This study evaluated the comparative efficacy of homologous and heterologous inactivated vaccines against that of the live Bacille Calmette–Guérin (BCG) vaccine on *M. bovis* isolation, gross TB lesions, and immune responses. Data from four controlled vaccination and challenge trials involving 41 calves were analysed. Animals were vaccinated with live BCG or heat-inactivated *M. bovis* via oral or parenteral routes, and immunologic and post-mortem analyses were conducted to evaluate vaccine performance. Compared with nonvaccinated controls, all vaccination strategies significantly reduced *M. bovis* bacterial loads in the lungs (up to 99%). However, bacterial loads in lymphoid tissues increased, confirming that tuberculosis is primarily a lymphatic disease. Diagnostic interference varied by vaccine type and administration route, with oral administration resulting in lower interference. Gross lesion scores were inconsistent across groups, suggesting limited utility as a measure of vaccine efficacy. Immune responses revealed increased detection of infection, particularly with inactivated vaccines. The lung bacterial load appeared to be decoupled from cellular immune responses, lymph node lesions and bacterial load, which were negatively correlated. Inactivated vaccines can offer a safe and effective means of reducing the bovine TB reproductive rate (R₀) even without total bacterial clearance. These findings highlight the need for revised evaluation criteria and support the integration of inactivated vaccines into TB control strategies.

**Supplementary Information:**

The online version contains supplementary material available at 10.1186/s13567-025-01637-2.

## Introduction

Animal tuberculosis (TB) is caused by species within the *Mycobacterium tuberculosis* complex (MTC), primarily *M. bovis* and *M. caprae*, which affect a wide range of hosts, including cattle. As TB has long been recognized as a zoonotic disease, eradication programs targeting cattle have been implemented in many countries. These programs have been largely successful, particularly in reducing TB prevalence in the primary zoonotic reservoir, dairy cattle.

However, in recent decades, the epidemiology of TB has proven to be more complex than previously understood. Beyond dairy cattle, other livestock, such as beef cattle, goats, pigs, and sheep, managed in extensive systems—often in contact with wildlife—serve as significant hosts. These hosts, which are frequently outside the scope of traditional cattle-centred control programs, play a critical role in maintaining MTC in the environment [1–3]. Additionally, wildlife hosts contribute significantly to the MTC maintenance community [4].

Compounding this challenge is growing evidence of the limited sensitivity of traditional diagnostic methods [5], highlighting the need for alternative TB control strategies. Among these alternatives, vaccination has emerged as a promising tool. However, cattle vaccination has been banned since the 1950 s because of concerns about interference with immune-based diagnostic tests used in eradication programs and doubts about its efficacy [6]. Notably, research conducted in Ethiopia has demonstrated the potential of cattle vaccination against TB. Even a nominal vaccine efficacy of 50% was shown to reduce the basic reproduction number (R₀) to less than 1, driving infection towards extinction at a significantly lower economic and social cost than test-and-cull strategies did [7].

Despite this, research efforts have predominantly focused on live BCG vaccines, while inactivated (killed) vaccines have been largely neglected [8]. The demonstrated success of inactivated vaccines in wildlife [9–11], their inherent safety, and insights gained from heat-inactivated paratuberculosis vaccines have rekindled interest in their use for TB control [12, 13]. Furthermore, the recent concept of trained immunity (TRAIM) [14] provides theoretical support for the use of vaccines. Despite early observations suggesting that TRAIM effects were exclusive to live vaccines [15, 16], the use of adjuvants in killed vaccines has shown potential to increase their infection-fighting ability. Additionally, oral administration of inactivated vaccines, which avoids eliciting specific immune responses [17], presents a promising opportunity for a *Differentiating Infected from Vaccinated Animals* (DIVA) strategy. This approach may protect against infection without interfering with official diagnostic tests.

Traditionally, tuberculosis is considered a respiratory disease because of its primary location in the thorax. However, in cattle, the lungs—histologically and functionally distinct from the lymph nodes—are less commonly affected. In Australia, for example, only 14.1% of gross lesions were located in the lungs, with 3.9% being single lesions [18]. In Spain, prior to the implementation of test-and-cull programs in the 1950 s, 48.8% of 1561 slaughtered cattle displayed gross lesions in the lungs [6]. In contrast, in the United States, no gross lesions were observed in a small sample of 15 cattle, although 6.7% were positive by isolation [19]. These findings, combined with evidence of immune response compartmentalization [20, 21], support the view of tuberculosis as a lymphatic disease with lungs as its exit (and entrance) portal [22] and suggest that giving the same weight to lymphatic infection as to lung infection may lead to an underestimation of the epidemiological risk in this slow-progressing, low-transmissibility disease [23], for which vaccination might reduce nasal shedding 100%, whereas immune tests only show a 60% reduction in prevalence [24]. Furthermore, the lower vaccine efficacy observed in experimental challenge trials [25, 26] than in field conditions [7, 27] may be partially attributed to differences in infection routes related to contact closeness associated with a housing-to-free ranging mode of life spectrum. In general, differences in vaccine protection studies can also be related to the large variability in experimental design [28–30], which makes it difficult to identify factors that modify vaccine performance. Differences in time and route of vaccination, as well as challenge methods that bypass natural infection pathways or short follow-up length, potentially bias efficacy assessments.

On the basis of these premises, we aimed to compare homologous and heterologous killed vaccines with the current standard live BCG vaccine. Our study evaluated these vaccines in terms of *M. bovis* isolation as the main indicator of transmission risk and gross TB lesions, specific immune responses, and DIVA performance as secondary indicators and discusses the factors affecting vaccine performance.

## Materials and methods

### Animals, vaccination, and sampling

This meta-analysis revisits data from four experimental vaccination and challenge trials involving a total of 41 calves. The results from these trials have been partially published in three reports [31–33].

#### First trial: vaccine diagnostic interference with a heterologous vaccine

Six Friesian calves, aged 2–3 months, were selected from farms in northern Spain with no known history of tuberculosis (TB) and shown to be negative by interferon-gamma release assay (IGRA). The calves were recruited at a feedlot and subcutaneously vaccinated with the commercial paratuberculosis vaccine Silirum*™* (CZ Vaccines, Porriño, Spain) (CPVP group).

**Figure 1:**
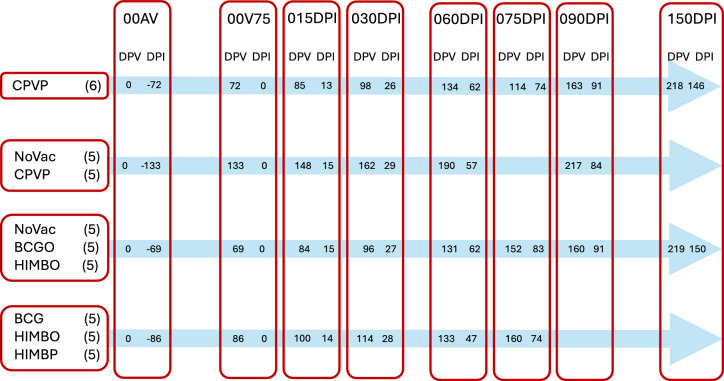
**Experimental design.** Study groups and experimental timeline. NoVac: Infected, nonvaccinated animals; CPVP: Parenteral commercial paratuberculosis vaccine; BCGO: oral BCG vaccine; HIMBO: oral heat-inactivated *M. bovis* (HIMB) vaccine; HIMBP: parenteral (intramuscular) HIMB vaccine. 00AV: Day 0 of the experiment, pre-vaccination; 00V75: standardized Day 75 post-vaccination; 015dpi: standardized Day 15 post-infection; 030dpi: standardized Day 30 post-infection; 060dpi: standardized Day 60 post-infection; 075dpi: standardized Day 75 post-infection; 090dpi: standardized Day 90 post-infection; 150dpi: standardized Day 150 post-infection. The numbers within the arrows indicate actual days post-vaccination (dpv) and post-infection (dpi) for each experiment.

After 1.5 months, the calves were transported to biosafety level 3 (BSL-3) facilities at NEIKER-BRTA. Following a 32-day acclimatization period, the calves were challenged endotracheally with approximately 10^6^ colony-forming units (CFUs) of a recent *M. bovis* isolate. Blood samples were collected from the jugular vein on Days −72, 0, 13, 26, 62, 74, 91, and 146 post-infection (dpi). Blood was drawn into lithium heparin-coated tubes for cellular immunity assays (Figure 1).

On Day 146, all calves were euthanized following standard approved procedures and subjected to full necropsy. Gross lesions were scored as described by Palmer et al. [34]. Samples were collected from seven lymph nodes, the tonsils, lungs, liver, and spleen for *M. bovis* isolation in both Coletsos solid media tubes and in the Mycobacterial Growth Indicator Tube (MGIT) system (Becton Dickinson, San Agustin de Guadalix, Madrid, Spain).

#### Second trial: vaccine diagnostic interference and protection with heterologous vaccines

This experiment is described in detail in Serrano et al. [31, 32]. Briefly, ten male Friesian calves, similar in characteristics to those used in Experiment 1, were randomly assigned to one of two groups: five calves received the commercial Silirum™ paratuberculosis vaccine (CPVP), and five calves were administered only phosphate-buffered saline (PBS) (NoVac).

After 110 days, all calves were transported to NEIKER-BRTA biosafety level 3 (BSL-3) facilities. Ten days after arrival, the animals were challenged endotracheally with *M. bovis*. Blood samples were collected on Days − 133, 0, 15, 29, 57, and 84 post-infection (dpi).

At 84 dpi, all the animals were killed and subjected to pathological and microbiological examination by seeding samples from the head (tonsils, nasal turbinate, and parotid, retropharyngeal and mandibular lymph nodes (LNs)), thorax (tracheal, tracheobronchial, mediastinal, pulmonary, and prescapular LNs), lung (right and left cranial and caudal lobes and medium and accessory lobes), abdomen (liver, spleen, and hepatic LNs), and others (prefemoral LNs) on solid Coletsos and liquid MGIT media.

#### Third trial: vaccine protection with live and inactivated homologous vaccines

These experimental results regarding the route of infection were published by Serrano et al. [33]. Fifteen calves were randomly assigned to one of three treatment groups:

NoVac: Nonvaccinated calves received 2 mL of plain PBS. BCGO: Calves received approximately 10^7^ CFUs of the BCG vaccine orally (*n* = 5). HIMBO: Calves received an oral dose of 10^7^ CFUs of heat-inactivated *M. bovis* (HIMB) vaccine (*n* = 5). This vaccine was produced with a wild boar *M. bovis* isolate (NEIKER strain #1403; SB0339) that was propagated and inactivated as described in previous experiments [10, 17, 25]. Calves were challenged with 10^6^ CFUs of NEIKER strain #1403 orally at 69 days post-vaccination (dpv). Finally, the calves were sacrificed at 150 dpi and subjected to necropsy and tissue sampling for mycobacterial isolation in Coletsos and MGIT. Samples were taken from the retropharyngeal, parotid, mandibular, tracheobronchial, prescapular, hepatic, mediastinal, jejunal and ileocecal lymph nodes and lung, liver, spleen and kidney.

#### Fourth trial: vaccine protection with live and inactivated homologous vaccines by different routes

To assess differences in protection and diagnostic interference related to antigen type, dose, and administration route, fifteen calves were randomly assigned to one of three treatment groups: BCGO: Calves received an oral BCG vaccine. HIMBO: Calves received 2 mL of an oral HIMB suspension containing 10^7^ CFUs of heat-inactivated *M. bovis*. HIMBP: Calves received 1 mL of a parenteral HIMB suspension containing 10^3^ CFUs of heat-inactivated *M. bovis*. This vaccine was formulated as a water-in-oil emulsion with 0.5 mL of Montanide™ ISA 50V2 adjuvant (Seppic, Paris, France) and 0.5 mL of PBS containing the inactivated bacterial suspension.

The animals were sacrificed at 74 dpi, and after gross pathological examination, samples were taken from the retropharyngeal, parotid, mandibular, tracheobronchial, mediastinal, prescapular, hepatic, popliteal and inguinal LNs and lung, liver, spleen and kidney for Coletsos and MGIT isolation.

### Challenge procedures

In trials 1, 2, and 4, all calves were challenged on Day 0 dpi via the endotracheal route with 2 mL of PBS containing approximately 10^6^ CFUs of an *M. bovis* field isolate. For trial #1, the strain used was PCR419 (03/2249), for trial #2, the strain used was 1403, and for the remaining trials, strain #08/2575 was used, all of which were field isolates. This last strain was determined to be SB0339, which was originally obtained from a naturally infected wild boar and has been used in previous studies [32, 33, 35]. Calves were sedated intramuscularly with 2% XILAGESIC® (10 mg/50 kg; Laboratorios Calier, S.A., Barcelona, Spain) prior to inoculation. The bacterial suspension was delivered with a syringe by inserting a needle between two consecutive tracheal rings (positions 25–30). Endotracheal air was aspirated to confirm placement before the inoculum was injected. In Experiment 3, the bacterial suspension was administered orally without sedation.

### Interferon-γ release assay (IGRA)

Blood was collected from the caudal vein into BD Vacutainer™ tubes containing lithium heparin (Becton Dickinson and Company, Sparks, MD, USA) (Figure 1). Lithium heparinized blood was aliquoted into cell culture plate wells for antigenic stimulation, which was initiated within 4 h of collection. The samples were incubated overnight at 37 °C in a 5% CO₂ incubator with different antigens and a nil control (Nil).

The antigens used included the following:

- Standard tuberculin: avian purified protein derivative (aPPD) and bovine purified protein derivative (bPPD) (CZ Veterinaria, Porriño, Spain).
- Protein complex: bPPD-derived protein P22 [36] kindly provided by Dr M. Dominguez.
- Defined antigens: Peptide cocktails ESAT6-CFP-10 and Rv3615c in trials #1 and #2, and ESAT-6 & CFP-10, Rv3615c and Rv3020c in trials #3 and #4, kindly provided by Drs G Jones and M Vordermeier [17, 37–40].

Both PPDs and P22 were used at a final concentration of 20 µg/mL, and synthetic peptides were used at 5 µg/peptide/mL.

Following incubation, the samples were centrifuged, and interferon-gamma (IFNγ) levels in the blood supernatants were measured with either the Bovigam™ TB kit (Thermo Fisher Scientific, Inc.) for the first and second experiments or the IDScreen® Ruminant IFNγ ELISA kit (IDvet, Grabels, France) for the third and fourth experiments, as per the manufacturers’ instructions. Optical densities (ODs) were measured at 450 nm with a Multiskan™ FC photometer (Thermo Scientific, Vantaa, Finland). No differences in reference Nil (*p* = 0.1703) or avian antigen (*p* = 0.9035) OD readings were found between the two kits; therefore, the results were treated without further kit differentiation.

For P22 and defined antigens, sample-to-positive (S/P) % values were calculated with the formula $([\left( {{\text{bPPD or p22 OD}}{-}{\text{aPPD OD}}} \right)/({\text{positive control}}\;\overline{{\text{X}}} {\text{OD}}{-}{\text{negative control}}\;\overline{{\text{X}}} {\text{OD}})]\, \times \,{1}00)$. The values were interpreted as described in Arrieta-Villegas et al. [41]. For the Bovigam test, a positivity cut-off of 0.1 OD difference between the sample and Nil control was used. For the IDScreen® Kit, a standard cut-off value of S/P% ≥ 16 was used to classify samples as positive for all antigens, including specific peptides.

IGRA assays for P22 and Rv3020c in the control group could not be conducted because of technical issues.

As shown in Figure 1, blood samplings were grouped into 8 standardized timepoints starting at vaccination (dpv) or at challenge (dpi) and designated according to the post-infection time. Both pre-challenge samplings were labelled “00”, with the added suffixes “AV” for pre-vaccination and “V75” for post-vaccination (standardized post-vaccination time).

### Skin test

At the last sampling, all calves in all the experiments were subjected to a cervical intradermal test with both the standard avian (2500 IU aPPD) and bovine (2500 IU bPPD) antigens in 0.1 mL doses and with two defined antigens (protein cocktails APHA1 (ESAT-6, CFP-10 and Rv3615c; 10 µg each protein) and APHA2 (ESAT-6, CFP-10, Rv3615c and Rv3020c; 10 µg each protein), kindly provided by Drs. G Jones and M Vordermeier) [40, 42–45]. Skin thickness was measured before injection and 72 h after inoculation. The results of the test for the increase in PPD skin thickness were interpreted in accordance with the official criteria (EU Council Directive 64/432/CEE and Spanish RD 2611/1996) for both the single intradermal test (SIT) and the comparative intradermal test (CIT). A calf was considered SIT positive when the increase in skin thickness at the bPPD site was 4 mm or greater. With respect to CIT, the animals were deemed positive when the increase in thickness of the bPPD injection site exceeded that of the aPPD site by more than 4 mm. With respect to the APHA reagents, the animals were considered APHA1- and APHA2-positive when the increase in skin thickness was equal to or greater than 2 mm.

### Clinical signs and post-mortem lesions

The animals were housed in NEIKER level 3 biosafety facilities and were fed a standard hay and concentrate diet with free access to an automatic drinking bowl. They had a daily overlayered straw bed and were supervised for clinical signs.

At the end of the experiment, immediately after the skin test, the animals were sedated (10 mg/50 kg 2% XILAGESIC®; Laboratorios Calier, S.A., Barcelona, Spain) and then euthanized (4–6 mL/50 kg T61; Intervet International GMBH, Unterschleissheim, Germany). Through a standard necropsy procedure, the calves were thoroughly inspected for TB-compatible lesions, and samples from the head (mandibular, parotid and retropharyngeal LNs and tonsils), thorax (prescapular, tracheobronchial and mediastinal LNs), lungs (right and left cranial and caudal lobes and medium and accessory lobes), abdomen (hepatic, jejunal and ileocecal LNs, liver and spleen) and other body areas (prefemoral and popliteal LNs) were collected for pathological and microbiological analyses. Both the isolation and gross lesion results were grouped according to tissue as the sum of the individual sample counts or scores for lung (exit portal), lymphoid, or other.

#### Gross pathology

The tissues were visually inspected, palpated and sliced in search of TB-compatible lesions. A previously described pathological scoring system was used for LNs, lungs and other organs [34]. LNs were scored as follows: 0, no visible lesions; 1, small focal lesion (1–2 mm); 2, several small foci; and 3, extensive lesions. Lungs and other organs were ranked as follows: 0, no visible lesions; 1, no external lesions but lesions detected upon slicing; 2, up to 5 lesions less than 10 mm in diameter; 3, more than 5 lesions less than 10 mm in diameter; 4, more than 1 distinct gross lesion larger than 10 mm in diameter; and 5, coalescing gross lesions. Histopathological evaluation was not used because it added very little in quantitative terms to gross pathology for a comparative study such as this one.

#### M. bovis isolation

LNs from the head, thorax, abdomen and carcass were collected with slight differences in each trial and included the mandibular, parotid and retropharyngeal pool, the tracheobronchial and mediastinal pool and the tracheal, tracheobronchial, mediastinal, mesenteric and hepatic LNs. The lymph nodes from the carcass usually included prefemoral and popliteal LNs. Lung samples (cranial, caudal and accessory lobes) were systematically collected for isolation with or without gross lesions. Any other lymph node, liver, spleen or kidney showing gross lesions was also sampled for isolation. Mycobacterial isolation was attempted simultaneously in Coletsos (Difco, Francisco Soria Melguizo SA, Madrid, Spain) and in BBL Mycobacteria Growth Indicator Tubes (MGITs) (Becton Dickinson, Franklin Lakes, NJ, USA) as previously described [10]. Two grams of sample was homogenized in 10 mL of sterile distilled water. The homogenate was separated into two 5 mL aliquots. One aliquot was processed for culture in supplemented MGITs (BACTEC MGITs supplemented with PANTA) following the manufacturer’s instructions. Five mL of 1.5% RonaCare cetylpyridinium chloride (Merck, Darmstadt, Germany) (w/v) was added to the remaining half of the homogenate, thoroughly mixed and incubated at room temperature for 12–18 h. After centrifugation (2500*g*, 5 min), the pellets were cultured in Coletsos tubes. MGIT tubes were incubated for 42 days in a BACTEC MGIT 960 System and Coletsos tubes at 37 °C for 4 months. The time to detection was transformed into CFUs according to an exponential power equation according to Sevilla et al. [46]. Tubes whose time to detection was longer than 15 days but shorter than 42 days (at the end of incubation) were assigned a value of 1 CFU. Tubes without growth within 42 days were scored as negative unless there was growth in the solid media, in which case a value of 1 CFU was assigned to that sample. The results of isolation were grouped according to tissue type (lymph node, lung and other locations), and the sum of the CFU count per tissue type for each animal was calculated and retained as the basic experimental measure for statistical analysis. DNA was extracted from all positive cultures for PCR confirmation [47] and spoligotyping of *M. tuberculosis*-positive samples [48]. The isolation results were ultimately grouped according to tissue into lymphoid (lymph nodes), lung (lung) and other (abdominal organs) for further analysis.

### Statistical analysis

Immunologic variables were analysed as dependent variables with a general linear model with treatment group and time as independent variables. To simplify and prioritize the global view, individual missing data for the experimental and control cells were filled with the corresponding previous control data. Differences between groups were assessed for each main effect and interaction versus the NoVac group with the corresponding treatment group post hoc comparisons. Isolation counts and gross lesion scores were used as the primary dependent variables. Although from an individual perspective, pathology is the most important outcome, from an epidemiological point of view, bacterial load is what truly determines population health. Therefore, all statistical testing focused on bacterial counts in both compartments, with an occasionally more detailed focus on the lung. The sum of all sample isolations was chosen because it was deemed to be the most representative and the one variable that allowed the best discrimination between the two variables of interest. Given the high variability of bacterial counts and lesion scores between experimental tissue and animals, the sums were corrected according to the ratio of each experimental mean to the fourth experimental mean. Bacterial counts and lesion scores were assessed according to a generalized linear model for tissue and treatment on the basis of the recommendation to use a negative binomial distribution model to assess zero inflated count results with equidispersion according to the quotient chi square by degrees of freedom overdispersion indicator (ODI) [49, 50]. To fall within the mathematical ranges of this type of distribution, all counts larger than 100 were transformed by division by 2000. This model had a greater reduction in error (R^2^) than the Gaussian and Poisson models did. Specifically, for CFU counts, the treatment and tissue Gaussian, Poisson and negative binomial models had R^2^ values of 0.1614, 0.3965 and 0.4538, respectively. Their ODIs were 105.6735, 13.3576 and 0.9601, respectively. Post hoc statistical pairwise comparisons were subjected to an uncorrected Student’s *t* test of significance when applied to vaccinated versus nonvaccinated controls or to the Bonferroni correction when applied to other comparisons [51]. Correlations between in vivo test (IGRA, Ab-ELISA, and SIT) results and isolation counts and lesion scores were calculated by both Pearson and Spearman correlation tests. Only correlations with coefficients of the same sign in both tests and a *p* < 0.1 were retained for discussion. Standard statistical significance was considered at *p* values < 0.05, but *p* values < 0.10 were also reported as potentially relevant but of lower significance because of low experimental units of groups. To more graphically describe the relationships among the immunological, microbiological and pathological variables, the final control results were subjected to principal component analysis (PCA) reduced to each one of the IGRA antigens (7 variables) and the microbiological (3) and pathological (3) variables by compartment. All the statistical analyses were carried out with the generalized linear model, GAMLj, and the factor and frequency modules of the jamovi application [51, 52].

## Results

### IGRA

**Figure 2:**
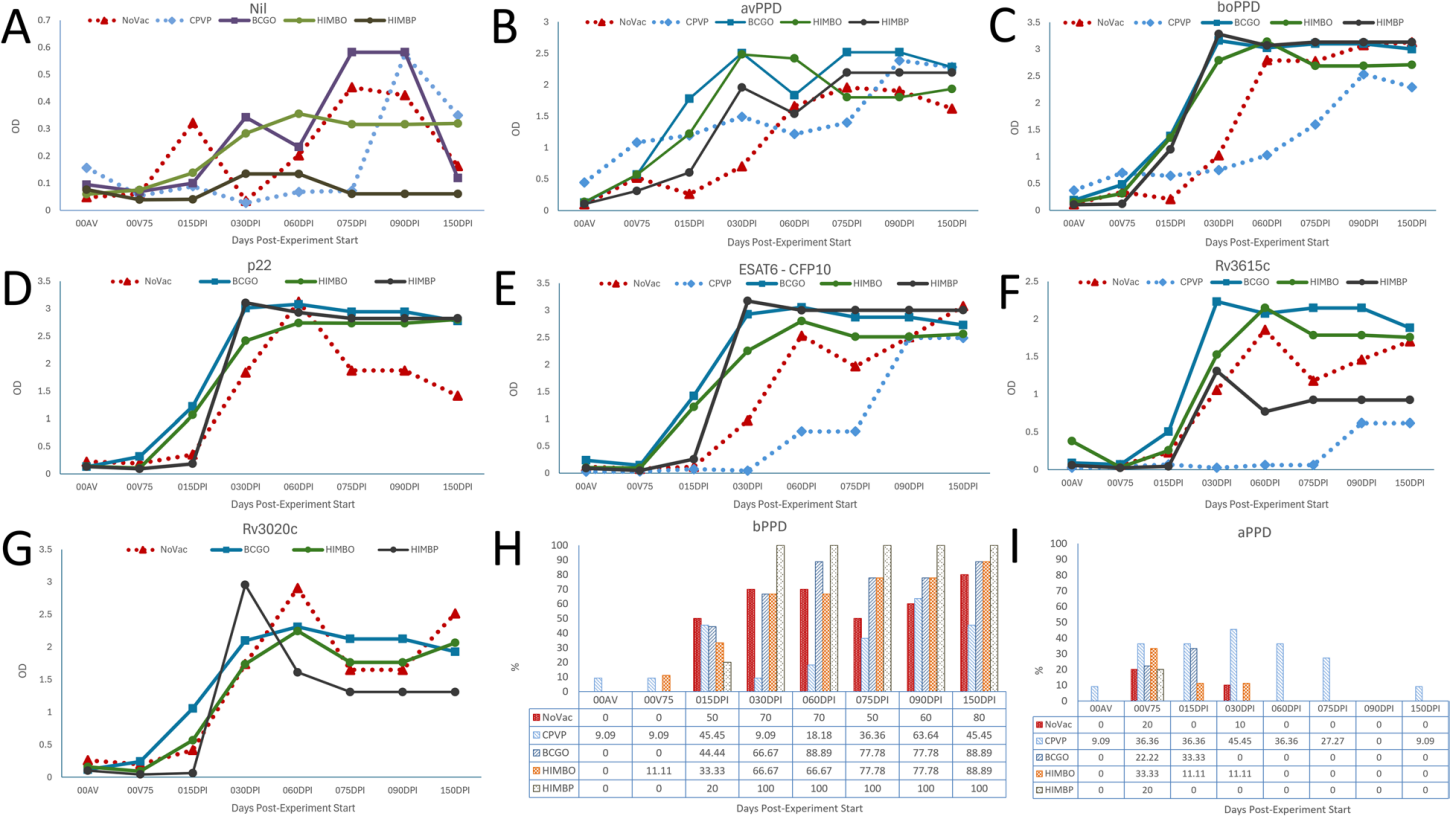
**Dynamics of the IFNγ release assay (IGRA) and specific antibody responses by group and time.** **A** IGRA with no specific antigen (Nil), only PBS (note: the y-axis scale is larger than that for the antigen responses); **B** IGRA with avian PPD tuberculin (avPPD); **C** IGRA with bovine PPD tuberculin (boPPD); **D** IGRA with p22 antigen (p22); **E** IGRA with ESAT6/CFP10 defined antigen (ESAT6-CFP10); **F** IGRA with Rv3615c defined antigen (Rv3615c); **G** IGRA with Rv3020c defined antigen (the CPVP group was not processed) (Rv3020c). **H** Frequency of positives in the standard IGRA diagnostic test with bovine PPD (bPPD); **I** PPD frequency of positives in the standard IGRA diagnostic test with avian PPD (aPPD). NoVac: Non-vaccinated control. CPVP: Parenteral commercial paratuberculosis vaccination. BCGO: oral BCG vaccination. HIMBO: Oral vaccination with heat-inactivated *M. bovis*. HIMBP: Parenteral vaccination with heat-inactivated *M. bovis*. The error bars have been omitted to avoid overlapping and overall cluttering. Instead, relevant differences are reported in the text. 00AV: Day 0 of the experiment, pre-vaccination; 00V75: standardized Day 75 post-vaccination; 015dpi: standardized Day 15 post-infection; 030dpi: standardized Day 30 post-infection; 060dpi: standardized Day 60 post-infection; 075dpi: standardized Day 75 post-infection; 090dpi: standardized Day 90 post-infection; 150dpi: standardized Day 150 post-infection.

The dynamics of the immune response in the IFNγ release assay with different antigens, including the Nil control, are shown in Figure 2.

Panel A in Figure 2 shows the levels of IFNγ in unstimulated blood. Overall, only the time of sampling had a statistically significant effect on the basal blood IFNγ levels (*p* = 0.0009), whereas neither treatment (*p* = 0.1963) nor interaction (*p* = 0.2851) effects were significant. The control group showed peaks at 15 dpi, 75 dpi and 90 dpi that were marginally significantly different from the initial mean (*p* = 0.0654, *p* = 0.0581 and *p* = 0.0357, respectively) and the other groups’ means (*p* = 0.1272, 0.0637, 0.1840, and 0.1236 for the BCGO, CPVP, HIMBO and HIMBP groups at 15 dpi; *p* = 0.3482, 0.1816, 0.6238 and 0.1349 on 75 dpi; and *p* = 0.4616, *p* = 0.0724, *p* = 0.5153 and *p* = 0.09621 on Day 90, respectively). The IFNγ means in the vaccinated groups also increased slightly between 75 and 90 dpi and were significantly different from those in the pre-vaccination control group (*p* = 0.0113 BCGO, *p* = 0.0237 CPVP, 0.0066 HIMBO, *p* = 0.030 HIMBP). These increased levels of basal IFNγ in untreated blood suggest an active immune response following challenge in the animals at these timepoints. With respect to diagnostic antigens, time of sampling had a main effect on the aPPD IGRA (*p* < 0.0001) (Figure 2, panel B), followed by interaction (*p* = 0.0293) and treatment (*p* = 0.0401). No differences were found before challenge in any of the groups (BCG *p* = 0.2342, CPVP *p* = 0.0536, HIMBO *p* = 0.2433, HIMBP *p* = 0.6860, NoVac *p* = 0.2401) compared to each group’s first control or compared to the NoVac group (BCGO *p* = 0. 9378, CPVP *p* = 0.3296, HIMBO *p* = 0.9158, HIMBP *p* = 0.9866). After vaccination and challenge, all groups significantly increased their aPPD IGRA mean readings, which differed only from those of the NoVac group at 30 dpi (BCGO *p* = 0.0005, CPVP *p* = 0.0028, HIMBVO *p* = 0.007, HIMBP *p* = 0.0887). Both vaccination and time of control were highly significant effects (*p* < 0.0001), as was their interaction (*p* = 0.0014) with respect to the bPPD IGRA (Figure 2, panel C). No significant differences were observed in the two pre-challenge controls (BCG 0.3565, CPVP *p* = 0.2741, HIMBO *p* = 0.6092, HIMBP *p* = 0.9770. NoVac *p* = 0.4409) or relative to the NoVac group (BCG *p* = 0.80312, CPVP *p* = 0.3860, HIMBO *p* = 0.8935, HIMBP *p* = 0.9896). Afterwards, all groups had significantly increased means with respect to their initial readings, and no differences between groups were observed until the end of the experiment except for the CPVP groups, which, starting at 30 dpi, had decreased readings relative to those of the NoVac group (BCGO *p* = 0.0567, CPVP *p* = 0.0118, HIMBO *p* = 0.4567, HIMBP *p* = 0.0554 *p* < 0.0001, *p* = 0.0104, *p* = 0.0538). The more specific antigens behaved similarly. For p22 (Figure 2, panel D), there were no significant differences in the pre-challenge controls between the BCG (*p* = 0.7823) and HIMB (*p* = 0.8441) groups (no CPVP group for this antigen) and the NoVac group (> *p* = 0.8402). At 15 dpi, the BCG group had larger values than the NoVac group did (*p* = 0.0512), whereas the HIMBO and HIMBP groups did not differ (*p* = 0.1091, *p* = 07417)). From then on, the BCGO (*p* = 0090) and HIMBP (*p* = 0.0127) groups, but not the HIMBO group (*p* = 0.1951), had higher values at 30 dpi, whereas differences disappeared at 60 dpi (BCGO *p* = 0.9105, HIMBO *p* = 0.3826 and HIMBP *p* = 0.6938) and increased at the last two controls (BCGO *p* = 0.0171, HIMBO *p* = 0.0545 and HIMBP *p* = 0.0610, and at 90 dpi and BCGO *p* = 0.0003, HIMBO *p* = 0.0002 and HIMBP *p* = 0.0015, at 90 dpi). For ESAT/CFP (Figure 2, panel E), no significant differences were observed in the first control (0.7086 > = *p* < = 0.9645) between the NoVac group and the vaccinated groups. At 15 dpi, both the BCGO (*p* < 0.0001) and HIMBO (*p* = 0.0008) groups had higher readings than the NoVac group did, which was not different from the CPVP (*p* = 0.9121) and HIMBP (*p* = 0.7126) groups. Afterwards, the BCGO, HIMBO and HIMBP groups (*p* < 0.0001) had higher readings than the NoVac group, which had higher readings than CPVP group (*p* = 0.0176) at 30 dpi. At 60 dpi and 75 dpi, the CPVP group remained below the NoVac group (*p* < 0.0001 and *p* = 0.0023), but BCGO did not differ from the NoVac group at 60 dpi, 90 dpi and 150 dpi (0 = 0.1085; *p* = 0.2605, *p* = 0.2887, respectively), although it was higher at 75 dpi (*p* = 0.0023). At this time, both the HIMBO (*p* = 0.948) and HIMBP (*p* = 0.0079) groups were higher. However, the differences disappeared in the last two controls (0.1152 < *p* < 0.9856). No significant differences in the Rv3615v IGRA (Figure 2, panel F) response were observed between the NoVac group (0.1682 ≥ *p* ≤ 0.9463) and the rest of the groups until 30 dpi, when that of the BCGO group peaked (*p* = 0.0059) and that of the CPVP group had not increased (*p* = 0.0414). The BCGO group maintained a higher level of response than that of the NoVac group but at variable levels of significance (*p* = 0.2908, *p* = 0.0051, *p* = 0.6181 and *p* = 0.4888, respectively). Similarly, the CPVP group maintained a level of response lower than that the NoVac group, including the last control (*p* = 0.0020, *p* = 0.0759, *p* = 0.0221 and *p* = 0.0058). The HIMB groups behaved quite differently, as the HIMBO groups had higher IFN responses than the NoVac control (*p* = 0.2175, *p* = 0.0496, *p* = 0.7234 and *p* = 0.3240), while the HIMBP groups had lower responses (*p* = 0.0920, *p* = 0.9622, *p* = 0.0466 and *p* = 0.0139). With respect to Rv3020c (Figure 2, panel G), no differences were detected among the BCGO, HIMBO and NoVac groups at any control (0.2030 ≤ *p* ≥ 0.9358) or between the HIMBP and NoVac groups except at 30 dpi (*p* = 0.0340), 60 dpi (*p* = 0.0238) and 150 dpi (*p* = 0.0354).

The IGRA response of the NoVac group increased starting at 30 dpi (*p* = 0.0172) and continuing at later timepoints (*p* < 0.0100). The differences were even greater in the BCG, HIMB and CPVP groups that also started showing statistically different mean values starting at 30 dpi (*p* < 0.0001) and continuing until the end when some convergence occurred (*p* < 0.0500). Panel I in Figure 2 shows the bovine tuberculosis diagnostic positive results according to the corresponding supplier algorithm. Only one animal in the CPVP group at the first control and another different one in the second (*p* = 0.5165 and *p* = 0.6338, respectively) were classified as positive by the IGRA in the first experiment. After challenge, positivity frequencies ranged between 20% and 50% in the 15 dpi control (*p* = 0.8087), whereas in the 30 dpi and 60 dpi controls, the HIMBP group reached 100% positivity, but the positivity of the CPVP group decreased to 9.1% and 18.2% (*p* = 0.0039 and *p* = 0.0040, respectively). This finding indicates that HIMBP experienced no diagnostic interference from 30 dpi onwards.

### Skin test

**Figure 3:**
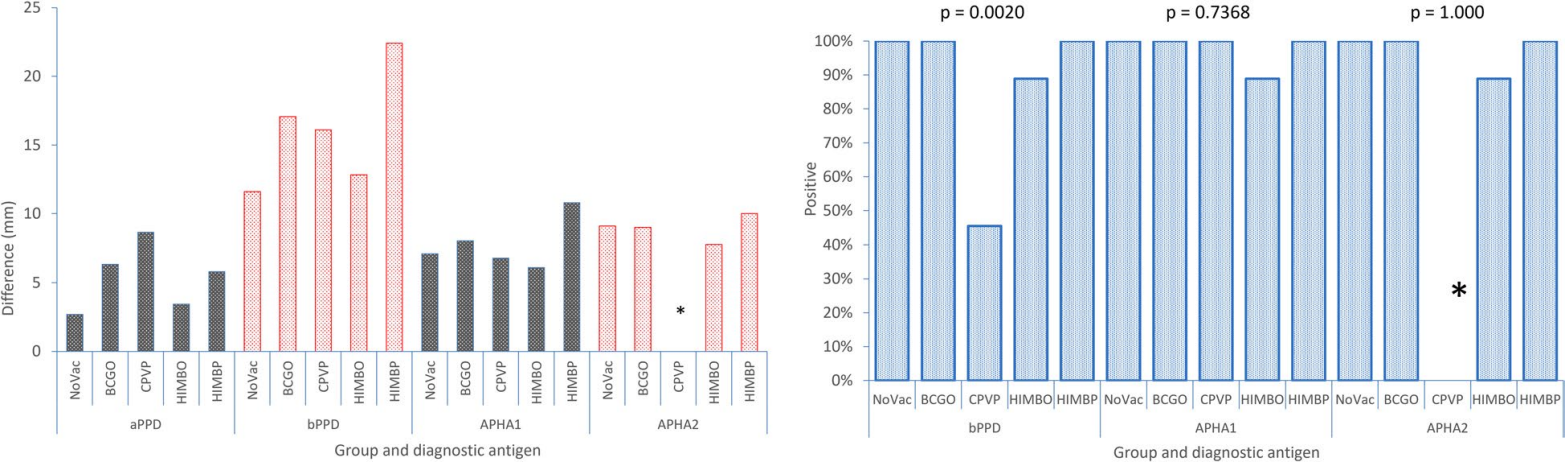
**Skin test results with different antigens.** aIDR: avian PPD; bIDR: bovine PPD; APHA1: protein cocktail 1 (ESAT-6, CFP-10 and Rv3615c); APHA2: protein cocktail 2 (ESAT-6, CFP-10, Rv3615c and Rv3020c). Only the BCG and HIMBP groups had 100% positive results in the bPPD comparative test. No group had 100% positive results with a comparative criterion and APHA1 antigen. APHA2, however, detected 100% of the nonvaccinated groups but failed to detect infection in the vaccinated groups. *APHA2 not tested for this treatment. NoVac: Nonvaccinated control. CPVP: Parenteral commercial paratuberculosis vaccination. BCGO: Oral BCG vaccination. HIMBO: Oral vaccination with heat-inactivated *M. bovis*. HIMBP: Parenteral vaccination with heat-inactivated *M. bovis.*

Panel A in Figure 3 displays the mean increases in the skin tests according to the type of vaccine and antigen at the last control. HIMBO was the group with the lowest increase among the vaccinated groups, with significant difference from the other vaccinated groups but not from the nonvaccinated control group with the bovine antigen. No significant differences were observed with APHA1- and APHA2-defined antigens compared to the NoVac group, except for HIMBP group, which showed a significantly larger increase with the APHA1. Panel B depicts the diagnostic interpretation of the skin test results according to current European legislation for its comparative version (bovine PPD) and to experimental single readings for APHA1 and APHA2. The CPVP group had the lowest frequency of positives, followed by HIMBO, whereas the other vaccine types reached 100% sensitivity (*p* = 0.0021).

### Clinical signs, post-mortem lesions and bacterial burden

**Figure 4:**
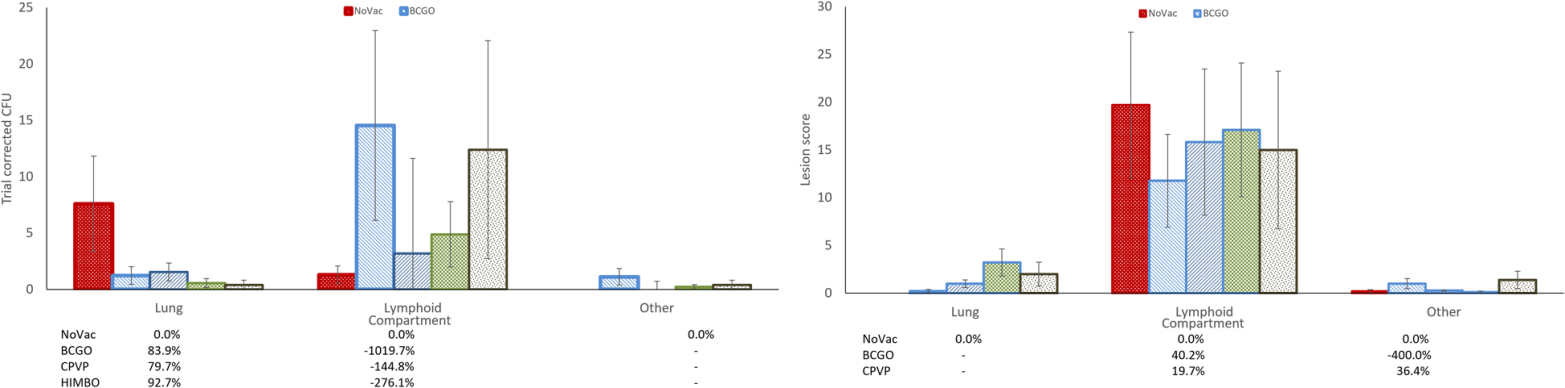
**Plot of** ***M. bovis* CFU and gross lesion score group means (with SEM) and reduction relative to NoVac according to treatment and compartment**. CFU and lesion score percent reduction below the compartment label. Note the strong interaction for isolation of the NoVac groups versus the vaccinated ones. NoVac: Nonvaccinated control. CPVP: Parenteral commercial paratuberculosis vaccination. BCGO: Oral BCG vaccination. HIMBO: Oral vaccination with heat-inactivated *M. bovis*. HIMBP: Parenteral vaccination with heat-inactivated *M. bovis.*

No clinical signs were observed after vaccination or challenge. All but five animals presented gross lesions compatible with TB in at least one location (one each from BCGO, CPVP and HIMBO and two from NoVac), and ten did not yield any *M. bovis* isolation (one from the BCGO group, six from the CPVP group, one from the HIMBO group and two from the NoVac group). The two negatives, both for gross lesions and for isolation, belonged to the BCGO and CPVP groups. The mean scores and SEMs of the pathological and microbiological variables for each group and tissue are shown in Figure 4. Both main effects, treatment (*p* = 0.0140) and tissue (*p* < 0.0001), and their interaction were significantly different (*p* = 0.0007) for isolation. For lesion score, treatment (*p* = 0.3113) was not statistically significant, but tissue (*p* < 0.0001) and their interaction (0.0796) were (Figure 4). This implies that vaccination induces different effects depending on the tissue. Taken together, all the vaccinated groups except the HIMBO group had higher bacterial counts than the nonvaccinated controls (data not shown). However, there was a much lower mean count in lung for all the vaccinated groups, accounting for between 84% and 95% reductions in the lung, and 1019%, 144%, 276% and 855% increases (BCGO, CPVP, HIMBO and HIMBP groups, respectively) in the lymphoid tissue. Thus, there was a large reduction in counts in the lung associated with vaccination, whereas there was a substantial increase in counts in the lymphoid tissue. In contrast, the lesion score in the vaccinated groups was higher in the lung than in the NoVac controls, whereas this group had higher values than the vaccinated group did in the lymphoid compartment (Figure 4).

**Figure 5:**
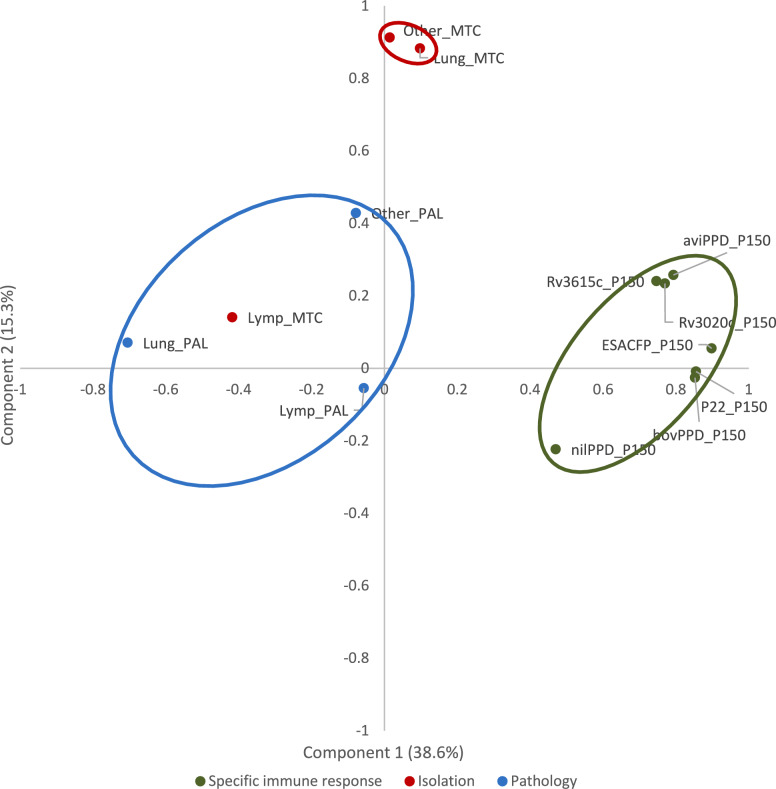
**Principal component analysis of the final control IFNγ results with each antigen, gross lesion score and bacterial load according to tissue.** The alignment of the immune response in the IGRA with different antigens with lesions and the lymphoid tissue bacterial load defines component 1 (38.6% variability) and demonstrates their decoupling from the bacterial load in lung and other tissues. Lung_MTC: *M. bovis* isolation from the lung; Lymp_MTC: *M. bovis* isolation from the lymph nodes. Other_MTC: *M. bovis* isolation from other tissues. Lung_PAL: Lung lesion score; Lymp_PAL: lymph node lesion score. Other_PAL: Other tissue lesion score. aPPD_P150: avian PPD at 150 dpi; bPPD_P150: Bovine PPD at 150 dpi; nilPPD_P150: Nil antigen at 150 dpi; ESACFP_P150: ESAT6-CFP10 defined antigen at 150 dpi; p22_P150: p22 antigen at 150 dpi; Rv3615c_P150: Rv3615 defined antigen at 150 dpi; Rv3020 defined antigen at 150 dpi.

The poor results of trial #3 did not allow a challenge route analysis regarding vaccination effects, as no isolations from the lung of the reference NoVac group were obtained in this experiment, which was the only one where this route was used (Figure 5).

### Correlation analyses

No clear pattern appeared after the correlation analysis of the individual immune test results with the post-mortem pathological and microbiological results was performed (Table 1). Considering all the groups together, only 9 correlations out of 224 fulfilled the selection criteria (both Pearson and Spearman of the same sign and with a *p* < 0.1). All were negatively related to lesions except for one that correlated an early ESAT6-CFP10 response at 15 dpi with bacterial counts in the lung. Four related specific and generic *M. bovis* antigen reactions were detected at late samplings with lung lesions, and some were lost when other nonlymphoid locations were included. The treatment groups seemed to behave differently, with each group showing many correlations that were lost when treated together in the all-group analysis. In the nonvaccinated controls, only the correlation between Rv3615c and bacterial isolation in the lymphoid tissues was significant, whereas in the vaccinated groups, the IGRA post-challenge responses to different antigens tended to correlate with lesion scores. Notable exceptions were the correlations of basal IFNγ, aPPD and bPPD at the first control of the BCGO group. Since no treatment had been applied at that time, this could be interpreted that nonspecific reactivity at vaccination might interact with bacterial multiplication at the end of the experiment. The CPVP group showed few correlations that were somewhat contradictory. Even though the correlation of the aPPD with the bacterial load correlated negatively both in lung and in lymphoid tissues by 90 dpi, there was a positive correlation between the basal IFNγ concentration at 90 dpi and the bacterial load in the lung. This group did not show any correlation between the immune response and lesion score. The HIMBO-vaccinated group showed a positive relationship between one of the specific antigens and the lung bacterial load at 30 dpi. This correlation was even more stable for the basal IFNγ levels, since it appeared by 30 dpi and persisted until the end of the experiment.

**Table 1:** **Correlations between IGRA and IDR results and post-mortem isolations and lesions according to location and compartment**

	Lung						Other					
	CFU			Lesion score			CFU			Lesion score		
	A/T	r	p	A/T	r	*p*	A/T	r	P	A/T	r	p
All groups	–	–	–	p22/60	− 0.8985	< 0.0001	ESCF/15	0.3532	0.0296	–	–	–
	–	–	–	Rv3020/60	− 0.5333	0.0035	–	–	–	–	–	–
	–	–	–	bPPD/90	− 0.8076	< 0.0001	–	–	–	–	–	–
	–	–	–	bPPD/150	− 0.6752	< 0.0001	–	–	–	–	–	–
NoVac	–	–	–	–	–	–	–	–	–	–	–	–
BCGO	Nil/00	0.7834	0.0125	–	–	–	–	–	–	Rv3615/30	− 0.7466	0.0208
	aPPD/00	0.9607	< 0.0001	–	–	–	–	–	–	–	–	–
	bPPD/00	0.8494	0.0038	–	–	–	–	–	–	–	–	–
	bPPD/60	0.689	0.0401	–	–	–	–	–	–	–	–	–
CPVP	Nil/A75	0.6965	0.0173	–	–	–	–	–	–	–	–	–
	aPPD/90	− 0.7214	0.0122	–	–	–	–	–	–	–	–	–
HIMBO	RV3020/30	0.7529	0.0192	p22/60	− 0.9404	0.0002	Nil/30	0.8671	0.0025	–	–	–
	–	–	–	Rv3615/30	− 0.7824	0.0127	Nil/60	0.8181	0.007	–	–	–
	–	–	–	bPPD/30	− 0.723	0.0277	Nil/75	0.7632	0.0167	–	–	–
	–	–	–	bPPD/75	− 0.9892	< 0.0001	Nil/90	Missing	Missing	–	–	–
	–	–	–	bPPD/90	Missing	Missing	Nil/150	0.7616	0.0171	–	–	–
	–	–	–	aPPD/150	− 0.7389	0.0229	AP2Dif	0.7669	0.0159	–	–	–
	–	–	–	bPPD/150	− 0.9883	< 0.0001	–	–	–	–	–	–
	–	–	–	ESCF/150	− 0.8624	0.0028	–	–	–	–	–	–
HIMBP	–	–	–	Nil/30	0.9068	0.0337	–	–	–	ESCF/00	0.9416	0.0168
	–	–	–	Nil/75	0.9599	0.0096	–	–	–	Rv3615/00	0.8452	0.0714
	–	–	–	Nil/90	Missing	Missing	–	–	–	Rv3020/00	0.8279	0.0834
	–	–	–	Nil/150	− 0.9118	0.031	–	–	–	Rv3020/15	0.8147	0.0931
	–	–	–	Rv3615/30	0.9895	0.0013	–	–	–	–	–	–

	Lymphoid						Nonlymphoid					
	CFU			Lesion score			CFU			Lesion score		
	A/T	r	p	A/T	r	p	A/T	r	*p*	A/T	r	p
All groups	–	–	–	ESCF/75	− 0.3696	0.0224	–	–	–	p22/60	− 0.8236	< 0.0001
	–	–	–	Rv3615/75	− 0.3374	0.0383	–	–	–	Rv3020/60	− 0.5803	0.0012
	–	–	–	–	–	–	–	–	–	–	–	–
	–	–	–	–	–	–	–	–	–	–	–	–
NoVac	Rv3615	− 0.8004	0.0054	–	–	–	–	–	–	–	–	–
BCGO	p22/150	− 0.9681	< 0.0001	aPPD/60	− 0.6815	0.0432	aPPD/00	0.8694	0.0011	Rv3615/30	− 0.6846	0.0419
	–	–	–	–	–	–	bPPD/00	0.9874	< 0.0001	–	–	–
	–	–	–	–	–	–	ESCF/00	0.8229	0.0035	–	–	–
	–	–	–	–	–	–					–	–
CPVP	aPPD/90	− 0.618	0.0427	–	–	–	Nil/A75	0.6965	0.0173	aPPD/A75	0.6863	0.0197
				–	–	–	aPPD/90	− 0.7214	0.0122	–	–	–
HIMBO	Nil/00	0.6948	0.0378	–	–	–	Rv3020/30	0.753	0.0192	p22/60	− 0.9311	0.0003
	–	–	–	–	–	–	–	–	–	Rv3615/30	− 0.7982	0.0099
	–	–	–	–	–	–	–	–	–	Rv3020/60	− 0.738	0.0232
	–	–	–	–	–	–	–	–	–	bPPD/75	− 0.9892	< 0.0001
	–	–	–	–	–	–	–	–	–	bPPD/90	Missing	Missing
	–	–	–	–	–	–	–	–	–	bPPD/150	− 0.9887	< 0.0001
	–	–	–	–	–	–	–	–	–	aPPD/150	− 0.7598	0.0175
	–	–	–	–	–	–	–	–	–	ESCF/150	− 0.8839	0.0016
HIMBP	bPPD/15	− 0.9924	0.0008	aPPD/00	0.9234	0.0251	–			bPPD/15	0.8737	0.0529
	bPPD/75	− 0.9118	0.031	–	–	–	–	–	–	bPPD/75	0.9479	0.0141
	bPPD/90	Missing	Missing	–	–	–	–	–	–	bPPD/90	Missing	Missing
	bPPD/150	Missing	Missing	–	–	–	–	–	–	bPPD/150	Missing	Missing
	–	–	–	–	–	–	–	–	–	–	–	-

A/T: Antigen and time, Skin test. aPPD: Avian PPD, bPPD: Bovine PPD, AP2Dif: APHA2 cocktail. IGRA. ESCF: ESAT6&CFP10 defined antigen, Rv3615: Rv3615 defined antigen, Rv3020: Rv3020 defined antigen.

### Principal component analysis

The factor analysis (Figure 5) clearly revealed that the specific immune response, as assessed by the IGRA test with different antigens, was strongly and negatively associated with the gross lesion scores, defining the first component of data variability that accounted for 38.6% of it. The lymphoid bacterial load also aligned with this axis in positive association with pathology in both lung and lymphoid tissue. Strikingly, bacterial burden in lung and other tissues was nearly at the 0 correlation with component 1 and nearly completely defined the second component (15.3% of the variability) along with gross lesions in other tissues and basal IFNγ levels.

## Discussion

The most significant finding of this study is the substantial reduction in lung bacterial load achieved through vaccination. This result highlights the importance of a frequently understated and even omitted variable that, however, provides a mechanistic basis for epidemiological evidence that vaccination can reduce the basic reproduction rate (R₀) below 1, leading to a progressive decrease in tuberculosis prevalence in vaccinated populations [7]. A similar observation has been reported for paratuberculosis, another relevant mycobacteriosis in ruminants [53]. This conclusion is partly attributable to a refined vaccine efficacy assessment strategy that differentiates between lymphoid and environment-interface compartments [22] and shifts the weight of TB damage evaluation from pathology to bacteriology and from semiquantitative to quantitative, further elucidating the complex pathogenesis of mycobacteriosis.

The experimental assays summarized in this study were designed to assess the immune mechanisms involved in the pathogenesis of *M. bovis* infection. They also evaluated sensitizations caused by various TB vaccine candidates, their delivery routes, potential interference with standard bovine tuberculosis diagnostic tests, and the protection offered by inactivated vaccines. This study tested several types of antigens (live and heat-inactivated homologues and heterologous inactivated antigens) and two delivery routes for inactivated vaccines.

Compared with nonvaccinated controls, all tested antigens and routes demonstrated the potential to elicit protective immune responses when challenged with a field strain of *M. bovis*. However, the nature of the response varied depending on the antigen, route, and parameters measured and even the target tissue compartment, occasionally leading to higher bacterial loads or lesion scores in the vaccinated groups and compartments. This variability underscores the need for tailored approaches to evaluate vaccine efficacy on the basis of intervention goals.

Unstimulated IFNγ levels in blood remained at baseline during the pre-challenge period but increased by Day 15 post-infection (dpi) across all groups for generic PPD antigens. Conversely, responses to defined antigens remained unchanged until 30 dpi, only reaching comparable levels across all groups by 60 dpi, with exceptions in the NoVac and BCGO groups for the ESAT-CFP and Rv3615 antigens, respectively. This finding indicates that some vaccines may not induce false positives without infection but may increase the detection capabilities of in vitro cellular immunity tests during early post-infection stages, particularly for PPD antigens. However, delayed responses to defined antigens may reduce the sensitivity of cells to immune-based tests.

Skin tests were evaluated only for sensitivity in this study, as they were performed solely at the final post-infection control. The standard bPPD appeared to benefit from vaccination, as significant differences in the increase in skin thickness relative to that in the NoVac control group were observed in all groups except the HIMBO group. The only difference observed with the defined antigen APHA1 was an increased mean thickeness in the HIMBP group, whereas no differences were noted with APHA2. The official bovine-avian comparative test appeared to perform satisfactorily, except with respect to the MAP vaccine and, to a lesser extent, to HIMB oral administration. In summary, vaccine interference was observed only as an increase in skin thickness, with no qualitative consequences in terms of sensitivity. However, fewer infected animals were detected among CPVP (36.4%) and HIMBO (77.8%) vaccinated animals with the standard comparative test. Only a minimal nonsignificant decrease in the frequency of positives in the HIMBO group with the defined antigens was recorded. This suggests that specific vaccinations with BCG or HIMBP were irrelevant compared to the official skin test responses in nonvaccinated animals. The use of defined antigens improved the sensitivity of the ST, as it allowed a single interpretation that cannot be used after avian sensitization with the paratuberculosis vaccine. Paratuberculosis infection and vaccination can decrease sensitivity in comparative tests, as they increase the avian response against which the bovine response is compared [54, 55].

Differentiating lung and lymphoid outcomes revealed distinct patterns of vaccination response, possibly related to pathological and immune responses that were decoupled from the bacterial load outside the lymphoid tissue according to the PCA. This might be an important observation that needs further attention, as it could substantially change tuberculosis pathogenetic mechanism models. Vaccination appeared to influence the bacterial load and lesion development in the lung but had a weaker effect on lymphoid tissues. This finding supports the tuberculosis model of a lymphatic disease with entry and exit points in the lungs [22]. While protecting the lungs may reduce transmission, bacteria may persist in the lymph nodes in a latent form. This dual effect supports a shift from eradication goals towards coexistence and slow control of transmission, as long as R₀ remains below 1 [7] and economic impacts are manageable.

Oral administration of HIMB was associated with lower IFNγ responses than parenteral administration, potentially indicating an anti-inflammatory effect. However, the specific immune responses varied on the basis of antigen type and delivery route, suggesting the need for tailored strategies to optimize protective efficacy.

Vaccination reduced lung bacterial loads but did not consistently affect gross lesion scores, challenging their reliability as a widely used measure of vaccine efficacy. Instead, focusing on bacterial load reductions in transmission-critical tissues such as the lung may provide a more accurate evaluation for epidemiological purposes. In this respect, our results do not fully agree with what has been reported in other studies in which the effects of vaccination were similar both in terms of lesions and bacterial loads, which generally do reasonably correlate. However, most of the vaccination studies were carried out with live BCG [30, 56–61] and reported isolation from either the lung [10, 56, 58, 62, 63] or the lymph node [64–66] or did not specify [24, 62]. Other studies skipped lung isolation and relied only on immune tests [67, 68] or pathological changes [69, 70]. This approach is valuable for human tuberculosis, where individual damage prevention is the central objective, but not for animal tuberculosis, where priority should be given to control the spread of the pathogen in the population. This perspective is supported by principal component analysis, which revealed a strong association of specific immune responses with lymph node damage and bacterial load along the first component but almost no association with lung bacterial burden, which determines the second component and to which the immunopathological variables do not appear to be linked. Thus, considering all the variables and compartments together might explain the inconsistent results in terms of vaccine protection. This might also have contributed to the early dismissal of killed vaccines [71] that, however, currently seems to be drawing interest from other researchers [11]. With respect to vaccine composition, antigens do not seem to play a major role, as the same product appears to suffer great variability across different studies [58, 59, 62, 67], and small variations in antigen specificity do not seem to greatly change the overall outcome [61, 72]. Finally, other key issues are the vaccination route and challenge route. The first might affect the way the bacteria are handled in the first moment of contact, which is critical, as earlier experiments on the quick destruction of radioactively marked bacteria suggest [71], and either induce an overstimulation of the inflammatory phenomena related to the acquired immune response or skip the natural innate barriers that mucosal immunity would normally provide. The use of challenge routes that bypass natural naso-pharyngeal innate nonspecific defence barriers likely leads to an underestimation of the protection afforded by local trained immune responses. Oral challenge, as previously demonstrated [33], can cause a reduction in infection efficiency and a shift towards digestive locations, preventing interpretation of the findings of this study and highlighting the need for more research on the interaction between vaccination and infection routes with respect to lung tuberculosis. Some long-term failures in response to natural exposure could also be explained by parenteral vaccination not having properly triggered the natural oro-nasal mucosa sensitization route [61–63, 73] or waning after a few weeks or months [24, 56, 68]. These possibilities are questions left open by the large variability in study design and target assessment. This highlights the need for a consensus in protection assessment to make studies comparable and cover the broadest range of factors that differentiate slow infections such as tuberculosis from acute infections whose mechanisms can influence outcomes. In that sense, we propose a reliance on quantitative microbiological variables, a separation of the lymphoid compartment from the lung and airways, an extension of the post-challenge follow-up to 1 or 2 years and, although not discussed here, additional attention to be given to the retropharyngeal lymph nodes that constitute both the oral and respiratory mycobacterial entrance/exit locations. This study confirms the discrepancies between the experimental and field outcomes observed in previous research [10, 74]. Field studies often report higher vaccine efficacy, likely due to lower infectious doses generated by lower transmission from vaccinated but infected animals and reduced animal-to-animal contact. However, in others, unexpectedly low protection has been observed [24, 65, 75]. This finding supports the need for revised evaluation criteria that better reflect real-world conditions.

These results suggest that vaccination induces epidemiological latency, confining bacteria to lymphoid tissues and reducing transmission. This aligns with recent findings by Fromsa et al. [7], who demonstrated additive effects of vaccination in preventing dissemination and increasing resistance in naïve individuals.

Wider adoption of vaccines, particularly those offering greater lung protection and having lower biological risks, could significantly reduce control program costs. Additionally, the nonspecific immune enhancement provided by these vaccines may reduce antibiotic usage and the associated risk of resistance development.

All these insights must be balanced against the limitations of this report, which can play both in favour and against a positive effect of vaccination. First, the variability caused by the different settings in each study might cover a wide range of external factors affecting tuberculosis pathogenesis and protection. However, it might also cause some relevant biases. Thus, Experiment 2 might have introduced a substantial distortion, as the experimental challenge resulted in very low rates of isolation and lesion scores that substantially decreased control reference values and maximized CPVP protection effects relative to those of the other vaccines. Another caveat is that the follow-up ranged between 2.5 and 7.5 months. Although this falls within most recent experimental tuberculosis studies in different species that use a follow-up period between 35 and 164 dpi [11, 25, 57, 76–78], which minimizes experimental expenses when dealing with a slow progressing disease with a high rate of latency, this follow-up might not fully represent long-term effects. In any case, it can generally be accepted that the first weeks of infection are a determinant of disease outcome by setting a path towards advanced pathology, latency or even full infection clearance [13, 27, 57, 76–78].

This study demonstrated that vaccination significantly reduced lung bacterial loads while exerting minimal effects on lymphoid tissues. Gross lesion evaluations alone are insufficient to measure vaccine efficacy, particularly for transmission risk. These findings support the hypothesis that vaccination induces compartmentalized immune responses, providing a mechanistic explanation for its efficacy in reducing transmission.

Effective control of chronic, multihost infections such as tuberculosis may benefit from shifting from eradication to strategies that strengthen barriers to spread and increase population-level resilience. Vaccination goals in animals should prioritize reducing transmission rather than total sterilization, especially in multihost ecosystems, in the initial stages and in those that have been shown to be refractory to eradication.

These findings underscore the potential of inactivated vaccines as biosafe and cost-effective tools for improving bovine tuberculosis control. They also highlight the need for revised evaluation methods to accurately assess vaccine efficacy in both experimental and field settings.

## Supplementary Information

**Additional file 1.** **Evolution of the immune response.** Individual bloodand skin test results for the experimental and control groups.

**Additional file 2.** **Post-mortem results.** Individual post-mortem gross isolation counts and gross pathology score results by tissue/compartment.

## Data Availability

The final data used for the evolution of the immune response (Additional file 1) and post-mortem findings (Additional file 2) analyses are included as two Excel spreadsheet files.
